# Effectiveness of neuromuscular electrical stimulation in severe acute pancreatitis complicated patients with acute respiratory distress syndrome: study protocol for a randomized controlled trial

**DOI:** 10.1186/s13063-023-07642-0

**Published:** 2023-09-21

**Authors:** Feng Zhou, Dingrong Fan, Yan Feng, Cuijuan Zhou, Xiaodong Chen, Xiaoyun Ran, Botao Tan

**Affiliations:** 1 Department of Rehabilitation Medicine, the Second Affiliated Hospital of Chongqing Medical University, 74 Linjiang Road, Chongqing, 40010 China; 2 Department of Pediatrics, the Second Affiliated Hospital of Chongqing Medical University, 74 Linjiang Road, Chongqing, 40010 China; 3 School of Nursing, Chongqing Medical University, Medical College Road, Yuzhong District, ChongqingChongqing, 400016 China; 4 Department of Critical Care Medicine, the Second Affiliated Hospital of Chongqing Medical University, 74 Linjiang Road, Chongqing, 40010 China

**Keywords:** Severe acute pancreatitis, Acute respiratory distress syndrome, Neuromuscular electrical stimulation, Function status

## Abstract

**Background:**

Severe acute pancreatitis complicated by acute respiratory distress is a common cause of intensive care unit (ICU) admission. These patients are at risk of a decline in physical activity due to bed rest. Neuromuscular electrical stimulation (NMES) has been recommended for ICU patients to strengthen muscles, but its effects on muscle atrophy, respiratory function, multiple organ dysfunction, and functional status of these patients remain to be proven.

**Methods:**

Patients (*n* = 80) will be prospectively randomized into an NMES group and a control group. The NMES group will receive NMES for 1 h per day for 7 days, and both the control and NMES groups will receive usual care. The efficacy will be assessed by an experienced physiotherapist and sonographer who will be blinded to the patient’s group assignment. Muscle power assessment (MRC scale), lower extremity circumference, grip strength, activities of daily living (Barthel index), and Marshall scores will be measured at baseline and posttreatment. The functions of the diaphragm assessments will be measured daily. Barthel index measurements will be followed up in the 1st month, 3rd month, and 6th month after discharge.

**Discussion:**

The trial will explore the effectiveness of NMES in functional status and diaphragm function in patients with SAP complicated with ARDS. The results of this trial will provide strong evidence of the efficacy of NMES in treating SAP patients with ARDS.

**Trial registration:**

This trial has been registered at the Chinese Clinical Trial Registry, and the registry name is “Effectiveness of neuromuscular electrical stimulation in severe acute pancreatitis complicated patients with acute respiratory distress syndrome: study protocol for a randomized controlled trial,” URL: https://www.chictr.org.cn, numbered ChiCTR2300068995. Date of Registration: 2023-03-03.

**Supplementary Information:**

The online version contains supplementary material available at 10.1186/s13063-023-07642-0.

## Introduction

Acute pancreatitis (AP) is a common disease in clinical practice due to an inflammatory disorder of the pancreas [1]. Twenty percent of AP patients may develop systemic inflammation leading to organ failure, defined as “severe acute pancreatitis (SAP)” [2–4]. Previous studies have demonstrated that the lung is the commonly damaged target organ caused by early induction of systemic inflammatory response syndrome (SIRS) in SAP [5]. One third of patients with severe pancreatitis develop acute lung injury (ALI) and ARDS in the first week [6], and these complications are responsible for high mortality and ICU admission [7–9]. In addition, blood purification, respiratory support, and sedation are common treatments but cause long-term bed rest for SAP patients with ARDS. Patients who stay in bed can experience muscle atrophy in the early stage. Studies have shown that the rectus femoris cross-sectional area decreases rapidly during the first week of bed rest; furthermore, muscle atrophy is more pronounced in patients with multiorgan failure [10, 11]. Muscle wasting is associated with reduced muscle strength and exercise capacity. The decline in exercise capacity is related to poor quality of life, increased mortality, and financial burden on healthcare [12–14]. Early intervention to reduce muscle wasting during the first week of bed rest in patients with SAP complicated with ARDS is urgently needed.

Several protocols have been proven to be feasible interventions to prevent muscle wasting in ICU patients. However, early mobilization and physical exercise are limited due to sedation, dyspnea, increased intra-abdominal pressure, or blood purification for the acute phase of SAP. NMES is an effective and common intervention to prevent muscle atrophy in clinical practice. NMES sends electrical impulses transcutaneously to motor neurons, causing involuntary muscle contractions. Studies have revealed that NMES is a convenient, safe, and beneficial intervention for early immobilization in the ICU [15, 16]. In patients who prolonged bed rest and inactivity, some studies have found that NMES can reduce muscle atrophy and preserve muscle strength [17, 18]. Furthermore, NMES can preserve fiber cross-section (CSA) and higher gene expression for myosin heavy chains [19, 20]. However, other studies have found that NMES is not a useful adjunct to revert muscle wasting and muscle layer thickness, especially for acute patients in the ICU [21–23]. In addition, Gutiérrez-Arias et al. revealed that NMES may reduce the duration of invasive mechanical ventilation for ICU patients, but the mechanism of the impact of NMES on ventilator weaning is unclear [24]. At present, there is insufficient evidence indicating that NMES intervention results in clinically important changes in muscle strength, respiratory function, and functional status for SAP patients with ARDS.

This trial aimed to investigate the effectiveness of NMES on function status outcomes in patients with SAP complicated with ARSD and to investigate the effects of NMES on muscle atrophy, diaphragm function, multiple organ dysfunction, and quality of life after discharge.

## Method

### Study design

**Fig. 1:**
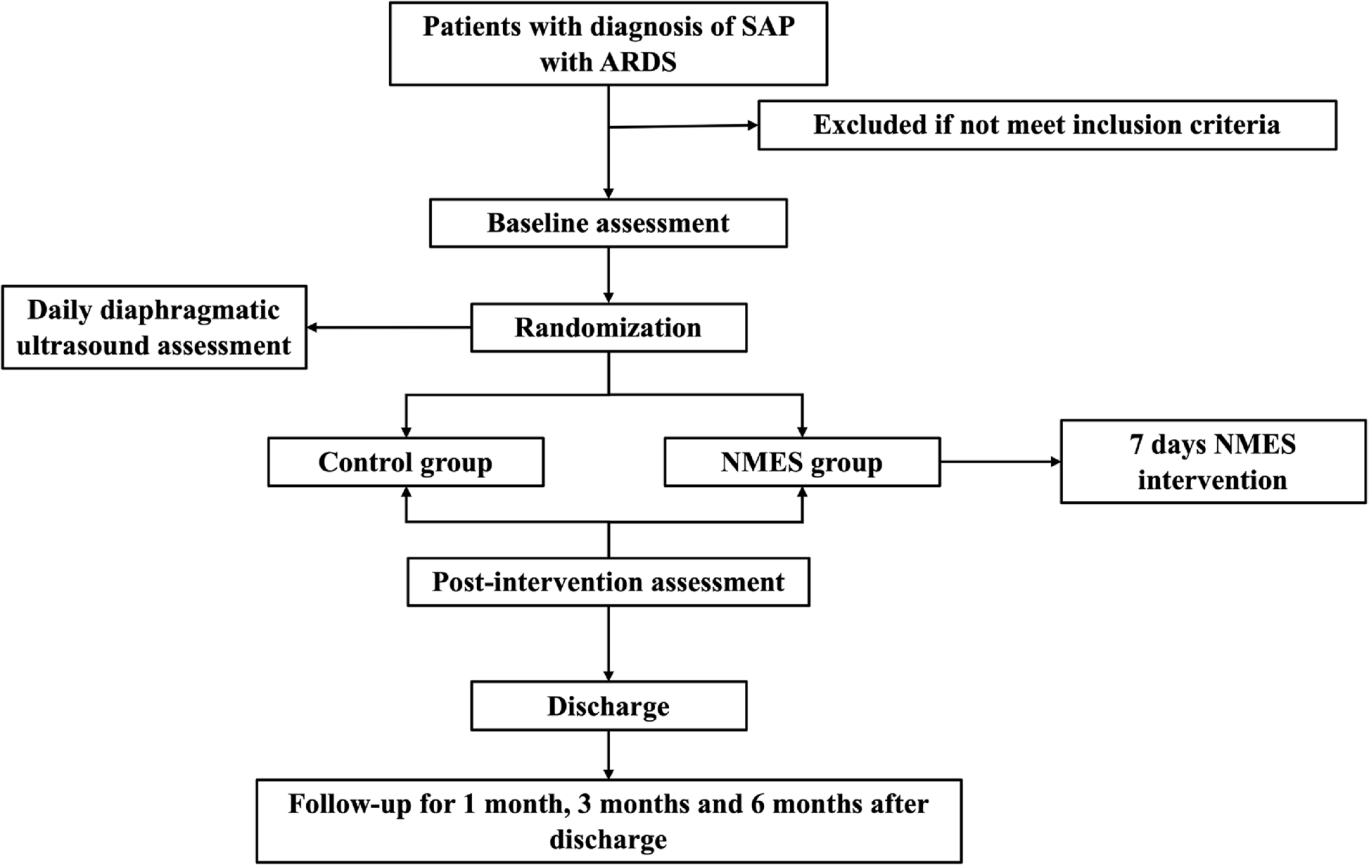
Flowchart of the study procedure

The overall objective of this trial is to explore the superior effects of NMES on preventing muscle wasting in patients with early SAP complicated with ARDS compared with sham intervention. We designed a randomized, prospective, single-blind trial (evaluator) because stimulation and contraction evoked during NMES therapy cannot be blinded to patients. Eighty patients who meet the inclusion criteria will be randomly divided into the NMES group and the control group at a ratio of 1:1. All participants will complete data collection and follow-up interviews. Assessment and treatment will be performed by a physician and therapist with 5 years of clinical experience, and diaphragm ultrasound evaluation will be performed by trained sonographers. Participants’ clinical assessments will be performed at baseline and 7 days postintervention, diaphragm ultrasound assessments will be performed daily, and follow-up assessments will be completed at months 1, 3, and 6 after discharge. The NMES group will receive neuromuscular electrical stimulation intervention and usual care for 7 days within 48 h of ICU admission, and the control group will receive the usual care only. The flowchart and study process are shown in Fig. 1 and Table 1, respectively.

**Table 1:**
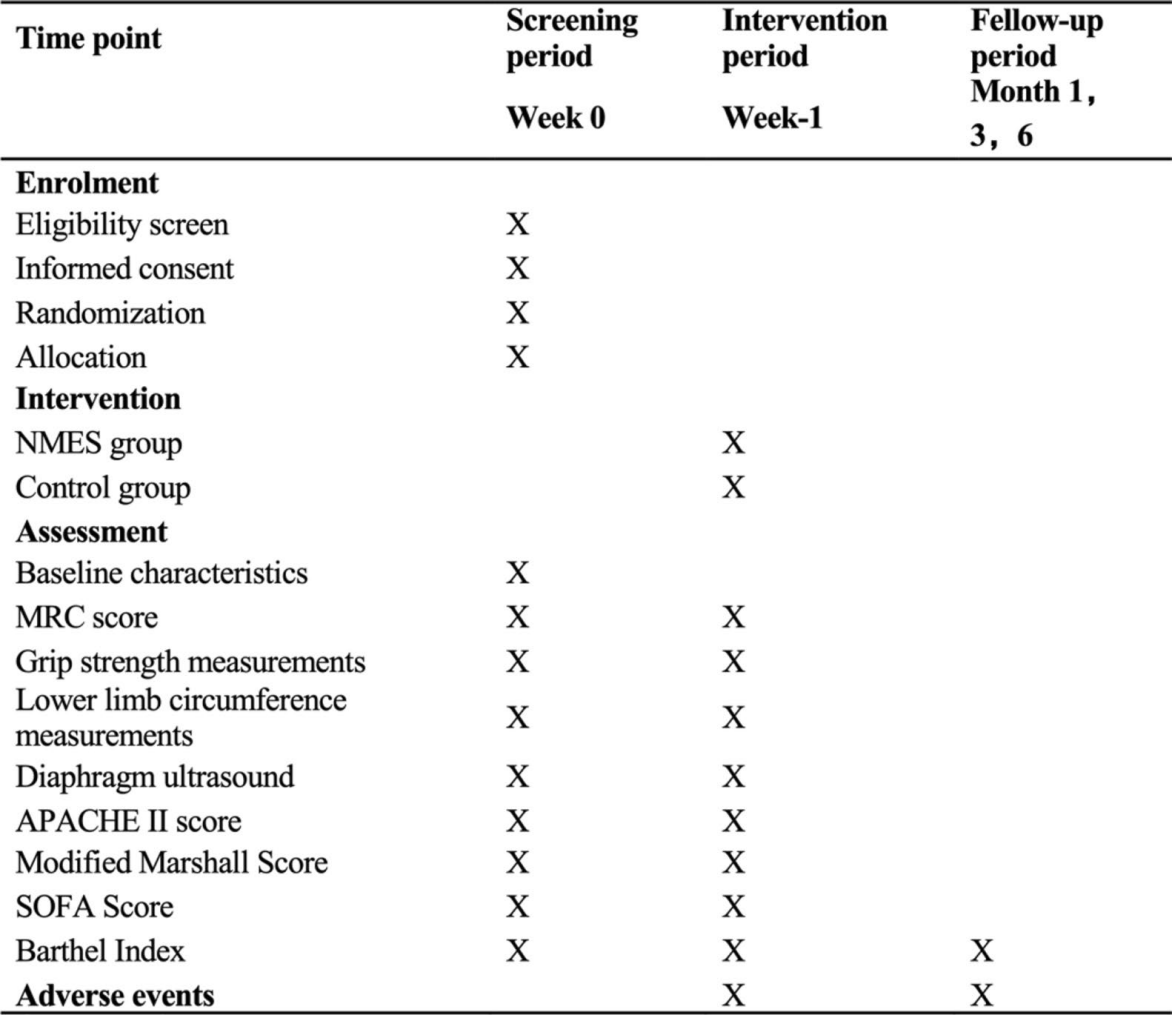
Study process of enrolment, intervention, and assessments

### Simple size

Campos DR et al. reported that the incidence of ICU-AW in the control group was P_1_ ≈ 0.45, and in the intervention group, it was P_2_ ≈ 0.11 [25]. According to those results, a sample size of 40 participants in each group was calculated to sufficiently detect the target effect size (0.5) with a type I error of 5% (*α* = 0.05) and 90% power (*β* = 0.10) by the PASS15.0 software. Allowing for a 20% loss to follow-up, this study was designed to include 80 cases.

### Inclusion criteria

- (1) Age 18 years or older
- (2) Time of admission to the ICU ≥ 24 h and diagnosis of SAP complicated with ARDS within 48 h of admission to the ICU

AP diagnosis requires two of the following three items: (1) persistent upper abdominal pain; (2) serum lipase and (or amylase) concentrations at least three times greater than the upper limit of normal; and (3) abdominal imaging test results show imaging changes consistent with AP.

SAP was diagnosed based on the revised Atlanta classification (RAC) in 2012 [26]. SAP is defined as persistent organ failure > 48 h. Organ failure is diagnosed as an organ score ≥ 2 for one of three organ systems based on the modified Marshall scoring system.

Diagnosis of acute respiratory distress (ARDS) syndrome was based on the Berlin criteria for acute respiratory distress syndrome. The required criteria must have all four of the following: (1) within 1 week of a known clinical insult or new or worsening respiratory symptoms; (2) chest imaging showing bilateral opacities not fully explained by effusions, lobar/lung collapse, or nodules; (3) respiratory failure is not fully explained by cardiac failure or fluid overload, need objective assessment (e.g., echocardiography) to exclude hydrostatic edema if no risk factor is present; and (4) oxygenation index (PaO_2_/FiO_2_) ≤ 300 mmHg under positive end-expiratory pressure (PEEP) or continuous positive airway pressure (CPAP) ≥ 5 cmH_2_O [27].

- (3) Signed informed consent.
- (4) Participant who voluntarily received this experimental treatment and completed the course of treatment.

### Exclusion criteria

- (1) Unable to walk independently before onset
- (2) Cognitive dysfunction before onset
- (3) Previous neuromuscular disorders (e.g., severe muscular atrophy, amyotrophic lateral sclerosis, myasthenia gravis, etc.)
- (4) Severe fractures, spinal cord injury, deep vein thrombosis, etc., need to be stopped
- (5) The onset time was ≥ 7 days, and the treatment was completed in the ICU of another hospital
- (6) Pregnancy
- (7) When the patient has a pacemaker
- (8) Death within 24 h
- (9) End-stage malignant tumor
- (10) The patient or his authorized client refused to participate in this study

### Participant recruitment

Patients meeting the eligibility criteria will be screened from the Department of Critical Care Medicine, the Second Affiliated Hospital of Chongqing Medical University. The recruitment period will be April 2023–April 2025. To screen SAP patients with ARDS, a physician in critical care medicine will complete the clinical diagnosis within 48 h of the patient’s admission and then will refer them to a physical therapist to assess whether the patient would be eligible to participate in this trial. Eligible patients will be randomly divided into the NEMS group or control group by an investigator. Baseline assessment will be completed after signing the informed consent form. Considering the risk of muscle wasting in the early stage of bed rest, patients will be given corresponding intervention immediately after participating in the trial. Investigators will check the list of eligible patients daily to determine whether the patients completed the assessment and intervention of the trial process. In addition, investigators will be responsible for maintaining long-term contact with patients and communicating about patient recovery, improving patient adherence, and reducing the failure rate of follow-up.

### Randomization

Numbers 1 to 80 will be randomly divided into the NMES group and control group using the SPSS 26.0 (IBM, USA) software by two investigators who were not involved in the trial. Another two investigators will make envelopes to hide groupings. The number can be seen on the surface of the envelope, but group information is inside the envelope. To ensure that the trial is blind, only investigators will have the authority to access envelope information. Eligible patients will be numbered in the order of the time they participated in the trial, and the investigators will open the envelope according to the patient number to determine the grouping of patients and further arrange for the patient to receive corresponding trial interventions.

### Blinding

This study was designed as a single-blind trial. Patient grouping, intervention, and assessment will be arranged by two investigators who are aware of the grouping. Assessors and statisticians will be blinded to the patient grouping. Because NEMS is electrical stimulation physical therapy, patients cannot be blinded. Unless an adverse event occurs, the blindness will not be broken until the end of the study.

### Intervention

Two physiotherapists with more than 5 years of clinical experience from the Second Affiliated Hospital of Chongqing Medical University will be responsible for the trial’s intervention. After undergoing standardized operation training, two qualified physiotherapists will administer NMES therapy to the NMES group. Meanwhile, the control group will receive standard usual care only. The intervention will last 7 days.

#### NMES group intervention

**Fig. 2:**
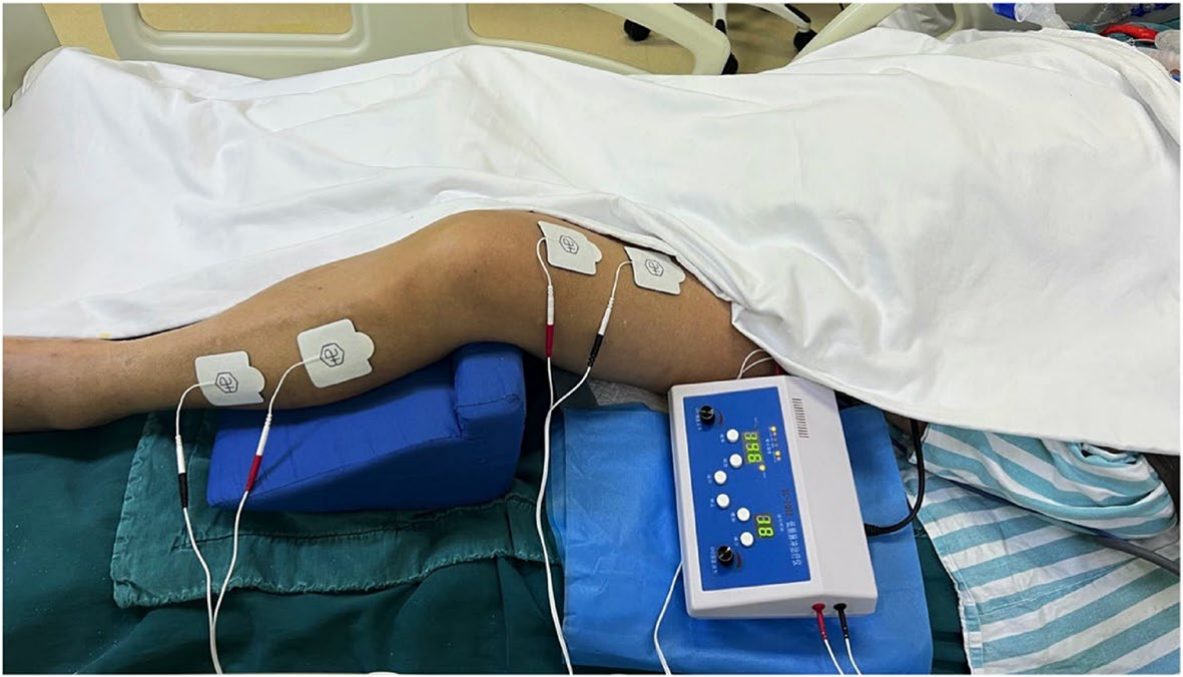
Intervention process of NMES for severe acute pancreatitis complicated patients with acute respiratory distress syndrome

NMES intervention will be performed on the first day of patient enrollment, and the intervention will continue for 7 days (Fig. 2). During the intervention, patients will take a supine position with the head elevated 30°, and the position of both lower limbs will be 15° hip flexion and 30° knee flexion to achieve the best muscle contraction [28]. Four groups of self-adhesive electrodes (5 cm × 5 cm) will be placed on motor points (rectus femoris, anterior tibial). The NMES intervention will be designed 60 min/day, including a 5-min warm-up, 50-min stimulation sessions, and a 5-min cooldown. The stimulation frequency of warm-up is 4 Hz, and the intensity will gradually increase to achieve visible contractions. Fifty-minute stimulation sessions consisted of 10 s of contraction stimulation (pulse width = 300 μs, frequency = 45 Hz), and the stimulation intensity met maximum muscle contraction without causing discomfort. In addition, a 5-s rest phase with a 4-Hz stimulation frequency will be performed. Cooldown sessions (frequency = 45 Hz) are composed of gradually decreasing intensity. During whole intervention sessions, intensity will be increased to avoid tissue accommodation. The physiotherapist will monitor the intervention period to prevent adverse reactions. In addition, NMES participants will receive routine care during the 7-day NMES intervention.

### Control group intervention

The control group will not receive NMES treatment. Routine care for the control group guided by treating clinicians, including health education, suction, regular turnover per 2 h, and elevating the head of the bed, will be completed by nurses. Early physiotherapy will be provided by physiotherapists. Patients who are unable to cooperate with sedation therapy will receive passive range-of-motion exercises. Responsive patients will be encouraged to receive out-of-bed mobilization and active exercise therapy (strength training, balance, standing, and walking). Early physiotherapy will be delivered according to physiotherapy intervention recommendations [29].

### Outcomes

#### Baseline characteristics

Participants’ baseline characteristics will be evaluated on the first day of enrollment. The following values will be recorded before the intervention: age, sex, duration of symptoms, respiratory rate, oxygen saturation, blood pressure, oxygenation ratio, abdominal circumference, intra-abdominal pressure, hemodialysis intervention, Marshall score, and APACHE II score. The baseline characteristics are shown in Table 2.

**Table 2:**
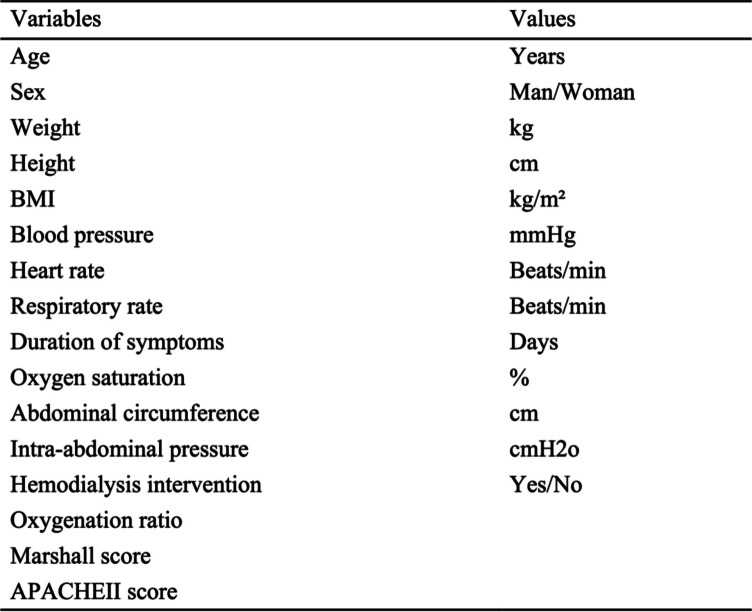
Patient’s characteristics

#### Primary outcome

##### MRC score

MRC score clinical assessments will be performed at baseline, and 7 days postintervention, MRC has proven to be a valid and reliable assessment scale to evaluate muscle weakness in critically ill individuals [30, 31]. MRC, as a convenient muscle strength assessment tool, is graded according to maximum muscle contraction, ranging from 0 to 5. The grade details are as follows: 0 = no contraction, 1 = visible and/or palpable contraction without limb movement, 2 = active movement, with gravity eliminated, 3 = active movement against gravity, 4 = active movement against gravity and submaximal resistance, and 5 = active movement against gravity and maximal resistance [32]. The MRC score was obtained by evaluating 6 groups of bilateral muscles. These include wrist dorsiflexors, elbow flexors, shoulder abductors, hip flexors, knee extensors, and ankle dorsiflexors. The total MRC score is 60, and higher MRC scores indicate better muscle strength [31].

#### Secondary outcomes

##### Grip strength measurements

Grip strength assessment will be performed at baseline and 7 days postintervention. Grip strength is a cost-effective clinical tool for muscle strength assessment. It is a strong predictor of sarcopenia. Additionally, lower weakness grip strength is associated with a higher risk of mortality [33]. Evaluators will use a CAMRY electronic hand dynamometer (EH101 Instruments, Guangdong, China) for grip strength assessment. In the bedside sitting position, participants will be asked to squeeze the dynamometer as hard as possible. The grip strength of each hand will be tested two times, and grip strength will be calculated through the average of the maximum values of the patients’ bilateral hands.

##### Lower limb circumference measurements

Lower limb circumference measurement assessments will be performed at baseline and 7 days postintervention. Trained evaluators will assess limb volume and appendicular skeletal muscle by measuring the patient’s lower limb circumference [34, 35]. The patient’s thigh and calf circumference dates will be measured with inelastic tape. Due to mobility limitations, patients will complete the circumference measurement in the supine position with hip and knee flexion. The thigh circumference will be measured midway between the inguinal fold and the base of the patella. Calf circumference will be measured at the maximum circumference between the knee and ankle. Both thigh and calf circumferences will be analyzed by adopting the mean of the left and right legs from the average of the two measurements for each leg.

##### Diaphragm ultrasound

Diaphragm ultrasound assessments will be performed daily. The diaphragm thickness and thickening fraction will be measured by ultrasound (LOGIQ Book, GE Healthcare, Waukesha, Wisconsin, USA). For patients in supine positions, diaphragm assessment will be performed using M-ultrasound with a 6–12 MHz frequency ultrasound probe at the 8th to 10th intercostal space of the right midaxillary line. The distance between the pleura line and peritoneum line will be measured as the thickness of the diaphragm (tdi) at the end-expiration. The thickness of the diaphragm will be obtained by averaging the measurements over three breathing cycles. In addition, the thickening fraction will be calculated as ((tdi end-inspiration − tdi end-expiration)/tdi end-expiration) × 100%. A low-frequency convex transducer is placed below the costal margin of the midclavicular line and anterior axillary line. Then, diaphragmatic excursion of the right hemidiaphragm will be recorded during quiet breathing.

##### APACHE II score

The APACHE II score assessment will be performed at baseline and 7 days postintervention. The APACHE II score is a severity-of-disease classification system for critically ill patients, with a total score ranging from 0 to 71 points. Higher scores correspond to a worse prognosis. The APACHE II score consists of the acute physiology score, age points, and chronic health points. The physiological variables of the Acute Physiology Score include body temperature, mean arterial pressure, heart rate, respiratory rate, A-a PO_2_ or PaO_2_ (for FiO_2_ ≥ 0.5 or < 0.5, respectively), blood pH or HCO_3_, serum sodium, serum potassium, creatinine, hematocrit, white blood cell count, and GCS. Acute Physiology Score was measured by nurses starting within 24 h of admission.

##### Modified Marshall score

Modified Marshall score assessment will be performed at baseline and 7 days postintervention. The modified Marshall score is an efficient classification tool for acute pancreatitis organ failure. The scale will rate respiratory status (PaO_2_ to FiO_2_), renal status, and cardiovascular status (systolic blood pressure, mmHg) on a scale of 0 to 4, with a score of 2 or above in any organ system indicating organ failure.

##### SOFA score

SOFA score assessment will be performed at baseline and 7 days postintervention. The SOFA score is traditionally employed in determining acute morbidity or the efficacy of clinical interventions in critically ill patients. The tool involves six criteria of respiratory, cardiovascular, hepatic, coagulation, renal, and neurological systems, in which each organ is scored on a scale of 0–4; the worse the organ dysfunction/failure, the higher the score.

##### Barthel Index

Barthel Index assessment will be performed at baseline and 7 days postintervention, and Barthel Index follow-up data will be collected in the 1st month, 3rd month, and 6th month after discharge. The Barthel index is used to assess the recovery of daily life function in critically ill patients, and it values ten items (personal hygiene, bathing self, feeding, toilet, stair climbing, dressing, bowel control, bladder control, ambulation, chair/bed transfers), yielding a score of 0–100. A higher score is associated with a greater degree of independence in daily life.

### Data collection and statistical analysis

Two trained evaluators will collect baseline data, outcomes, and follow-up data. Participant trial data will be recorded through the case report form. Participants will be informed of follow-up visits after discharge from the hospital, and follow-up data will be obtained through telephone questionnaires or home visits. Two investigators will check the case report form twice to ensure accuracy and completion and record the data of the case report form into the Excel file with double-check.

All participants can withdraw from the trial at any time. If participants terminate the trial or deviate from the intervention protocol, the investigators will record the trial data from the participant’s last intervention and report the reason for withdrawal.

The SPSS 26.0 software (IBM SPSS, Chicago, IL) was applied to analyze the baseline characteristics and outcomes between the two groups. *x̅* ± s will represent continuous variables that conformed to the normal distribution, and the *t* test will compare the differences between the two groups. The Mann–Whitney *U* test will be applied if continuous variables are not normally distributed. Categorical variables will be expressed as frequency (percentage, %), and the chi-square test will be used to compare the differences between the two groups. Effect estimates will be provided with corresponding 95% confidence intervals, and *P* values ≤ 0.05 will be considered significant. Aiming to preserve the unique benefit of randomization, regardless of whether that participant received the allocated intervention, the principles of intention-to-treat analysis will be used. All randomized participants will be included in the analyses, and data will be analyzed in the group in which the participants were originally randomized. All available data from the NMES group that was originally randomized will be analyzed as an intervention group, including participants from the NMES group who do not complete the NMES intervention or even do not receive NMES intervention. Similarly, all available data from the control group that was originally randomized will be analyzed as the control group, including any who receive the NMES intervention. Multiple imputation (MI) using the mice package in R will be applied to analyze the missing data.

### Safety assessment and monitoring

NMES intervention will be carried out strictly based on the instrument operation specifications and implemented by two physiotherapists who have completed operation training. Patients will be informed of the potential adverse events of NMES and sign an informed consent form. Adverse events may occur during treatment: mean arterial blood pressure < 65 mmHg, 40 bpm < heart rate < 130 bpm, SpO_2_ < 88%, skin redness, skin burns, skin blisters, pain, myocardial ischemia, and arrhythmia. The NMES group will complete NMES therapy under the supervision of a physiotherapist. During treatment, any patient discomfort will be dealt with immediately (reduce the intensity of electrical stimulation or stop the intervention). The research team will judge the severity of adverse events and analyze the correlation with the intervention. Patients who suffer harm or complications caused by the intervention will receive medical treatment at the Second Affiliated Hospital of Chongqing Medical University free of charge. All serious adverse events will be reported to the ethics committee within 48 h.

Based on the sequential study design, the primary outcome will be analyzed when 50% of participants (40 participants) have randomly enrolled to complete the intervention and data collection. An independent statistician who is blinded to the intervention process will be responsible for performing the data analysis. Type I error statistical analysis will be performed using the Pocock approach with *α* = 0.05. The interim analysis results will be reported to the data monitoring committee (DMC). The DMC will access all data and discuss to decide whether the trial should be continued, modified, or halted earlier than intended.

The data monitoring committee (DMC) will comprise intensive care physicians, rehabilitation physicians, rehabilitation therapists, and statistical experts. DMC members are independent of the sponsor and competing interests. The DMC is mainly charged with safety monitoring and the application of statistics. The DMC will monitor adverse reactions in the study to avoid increasing the risk to the patients. In addition, the DMC will review the data and monitor the quality of the trial conduct (protocol compliance, enrollment status, dropout rate of subjects, and data completeness) and assist the sponsor in deciding whether to terminate the trial early or modify the trial protocol. The DMC will check the quality and plausibility of the data every 2 months.

The adjustment plan of the research protocol (eligibility criteria, intervention plan and process, outcomes, and analyses) will be discussed by the research team. The revised protocol will be submitted to the ethics research committee (the committee) and the institutional review board (the board) of the Second Affiliated Hospital of Chongqing Medical University. For the quality of the study, any modifications to the protocol will be reviewed by the committee and the board.

### Ethics and dissemination

The study protocol was approved by the Hospital Research Ethics Committee of Chongqing Medical University on December 30, 2022 (approval no. 2022–301). This protocol was designed on December 30, 2022, with the version of V2.0. The study has been registered with the China Clinical Trial Center with the registration number (no. ChiCTR2300068995). Two researchers will be responsible for signing the informed consent of the participants. All participants will be informed about the benefits and risks of this trial and sign the informed consent by strictly voluntary consent. Participants can withdraw from the study at any time.

The patient’s data will be kept confidential, and only the researchers who manage the data will have access. The grouping information before the trial, informed consent, and the case report form will be kept by two researchers. Among them, two researchers will enter the paper version of the case report form data into an Excel file for preservation and encryption. Only two researchers will have access to the file. The patient’s paper case report form will be sealed in an envelope and stored in the locked file cabinet of the Department of Critical Care Medicine, the Second Affiliated Hospital of Chongqing Medical University. Except in the case of unblinding, only two investigators had access to patient information. According to the medical archive preservation rules and the principles of Good Clinical Practice (GCP), these data will be kept for at least 5 years and be used only for this study.

Participants and the public will not directly participate in the design and implementation of the study. Considering the patient’s privacy, the data will not be released directly to the public, but the protocol and results will be released. Meanwhile, the results of the experimental study will be presented and discussed at academic conferences, and experimental results will be published in peer journals.

## Discussion

To the best of our knowledge, this is the first study to focus on functional recovery, aiming to explore the effect of early NMES intervention on SAP with ARDS. The changes in muscle mass, diaphragm function, multiple organ dysfunction, and quality of life will be demonstrated after receiving 7 days of NMES intervention. In addition, the effect of early NMES intervention in the 1st month, 3rd month, and 6th month after discharge will be determined through follow-up.

ARDS is a common early complication of SAP, occurring 2–7 days after pancreatic inflammation. Furthermore, Fei et al. [36] indicated that ARDS increases the mortality rate of SAP in the first week of the disease. To ensure the stability of patients’ lives, critical care treatment is needed. Mechanical ventilation, blood purification, sedation, and analgesia are the supportive care for SAP with ARDS. However, critical care treatment impairs the patient’s physical activity, causing physical function to drop sharply in the early stage. A study of patient quality of life showed that survivors of SAP had significantly reduced SF-36 scores compared with age-matched controls, especially in the domains of social and physical function and general health [37]. Although there is currently insufficient evidence that SAP with ARDS will impair muscle mass, SAP and ARDS are major factors that attenuate muscle mass and reduce quality of life, respectively. Several studies have indicated that severe pancreatitis demonstrates a strong association with myosteatosis and muscle attenuation, which is related to poor survival [38, 39]. Dinglas et al. [40] showed that more than 1/3 of ARDS survivors experienced muscle weakness at hospital discharge, and 5-year follow-up outcomes after ARDS onset showed that patients with lower MRC sum scores were associated with higher mortality. The results of the 6-min walk test (6MWD) and SF-36 measures demonstrate that ARDS patients have reduced exercise capacity and quality of life in the first year [41]. To investigate the protocol for preventing physical function impairment, this trial aims to provide sufficient evidence about the efficacy of early NMES treatment in patients with SAP complicated with ARDS.

Considering the poor ability to participate in physical activities autonomously in early patients with SAP combined with ARDS, we plan to provide NMES intervention for 7 days within 48 h of admission to the intensive care unit. This study designed NMES to be applied in patients’ bilateral quadriceps and tibialis anterior for approximately 60 min/day. To assess the effect of NMES on muscle mass, the MRC scale will be used to evaluate muscle strength. In addition, we collected the changes in the latitude of the patients’ bilateral lower extremities and grip strength [42, 43]. Finally, whether NMES has a positive effect on diaphragm function and multiple organ dysfunction will be further explored through this trial. We will follow-up on quality of life to determine the sustained effect of NMES on patients with SAP and ARDS.

Although there are not enough studies on the efficacy of NMES for SAP with ARDS in the current era, recent evidence has shown that early NMES can reduce muscle atrophy and outcome measures for postoperative and critically ill patients. Campos et al. reported that compared with the early mobilization group, early NMSE and mobilization intervention improved the functional status score on the first day awake for patients who were mechanically ventilated [25]. A similar small-sample study showed that patients who underwent prolonged mechanical ventilation received NMES on the vastus lateralis and rectus femoris, concluding that the NMES group significantly increased MRC points and prevented decreased limb circumference [44]. To further investigate the therapeutic effect of NMES, we aimed to examine the effects of NMES in SAP patients with ARDS.

In conclusion, this trial compared the effect of early neuromuscular electrical stimulation on patients with SAP and ARDS through a randomized controlled trial, aiming to determine the effects of NMES on muscle strength, diaphragm function, multiple organ dysfunction, and quality of life.

### Supplementary Information

**Additional file 1.**

## Data Availability

Not applicable.

## Abbreviations

NMES: Neuromuscular electrical stimulation
AP: Acute pancreatitis
SAP: Severe acute pancreatitis
ARDS: Acute respiratory distress syndrome
ICU: Intensive care unit
MRC: Medical Research Council
APACHE II: Acute physiology and chronic health evaluation II
DMC: Data monitoring committee

## References

[1] Lee PJ, Papachristou GI (2019). New insights into acute pancreatitis. Nat Rev Gastroenterol Hepatol.

[2] Garg PK, Singh VP (2019). Organ failure due to systemic injury in acute pancreatitis. Gastroenterology.

[3] De Campos T, Deree J, Coimbra R (2007). From acute pancreatitis to end-organ injury: mechanisms of acute lung injury. Surg Infect (Larchmt).

[4] Werner J, Hartwig W, Uhl W, Muller C, Buchler MW (2003). Useful markers for predicting severity and monitoring progression of acute pancreatitis. Pancreatology.

[5] Zhu AJ, Shi JS, Sun XJ (2003). Organ failure associated with severe acute pancreatitis. World J Gastroenterol.

[6] Jacobs ML, Daggett WM, Civette JM, Vasu MA, Lawson DW, Warshaw AL, Nardi GL, Bartlett MK (1977). Acute pancreatitis: analysis of factors influencing survival. Ann Surg.

[7] Huang Y, Xiao J, Cai T, Yang L, Shi F, Wang Y, Li Y, Shi T, Li C, Peng Y (2019). Immature granulocytes: a novel biomarker of acute respiratory distress syndrome in patients with acute pancreatitis. J Crit Care.

[8] Jin YH, Liu Y (2019). Efficacy of blood purification for severe pancreatitis and acute respiratory distress syndrome. Medicine (Baltimore).

[9] Zhang W, Zhang M, Kuang Z, Huang Z, Gao L, Zhu J (2021). The risk factors for acute respiratory distress syndrome in patients with severe acute pancreatitis: a retrospective analysis. Medicine (Baltimore).

[10] Puthucheary ZA, Rawal J, McPhail M, Connolly B, Ratnayake G, Chan P, Hopkinson NS, Phadke R, Dew T, Sidhu PS (2013). Acute skeletal muscle wasting in critical illness. JAMA.

[11] Weber-Carstens S, Deja M, Koch S, Spranger J, Bubser F, Wernecke KD, Spies CD, Spuler S, Keh D (2010). Risk factors in critical illness myopathy during the early course of critical illness: a prospective observational study. Crit Care.

[12] Herridge MS, Tansey CM, Matte A, Tomlinson G, Diaz-Granados N, Cooper A, Guest CB, Mazer CD, Mehta S, Stewart TE (2011). Functional disability 5 years after acute respiratory distress syndrome. N Engl J Med.

[13] Van Aerde N, Meersseman P, Debaveye Y, Wilmer A, Gunst J, Casaer MP, Bruyninckx F, Wouters PJ, Gosselink R, Van den Berghe G (2020). Five-year impact of ICU-acquired neuromuscular complications: a prospective, observational study. Intensive Care Med.

[14] Schefold JC, Wollersheim T, Grunow JJ, Luedi MM, Z’Graggen WJ, Weber-Carstens S (2020). Muscular weakness and muscle wasting in the critically ill. J Cachexia Sarcopenia Muscle.

[15] Baron MV, Silva PE, Koepp J, Urbanetto JS, Santamaria AFM, Dos Santos MP, de Mello Pinto MV, Brandenburg C, Reinheimer IC, Carvalho S (2022). Efficacy and safety of neuromuscular electrical stimulation in the prevention of pressure injuries in critically ill patients: a randomized controlled trial. Ann Intensive Care.

[16] Segers J, Hermans G, Bruyninckx F, Meyfroidt G, Langer D, Gosselink R (2014). Feasibility of neuromuscular electrical stimulation in critically ill patients. J Crit Care.

[17] Gerovasili V, Stefanidis K, Vitzilaios K, Karatzanos E, Politis P, Koroneos A, Chatzimichail A, Routsi C, Roussos C, Nanas S (2009). Electrical muscle stimulation preserves the muscle mass of critically ill patients: a randomized study. Crit Care.

[18] Hardy EJ, Hatt J, Doleman B, Smart TF, Piasecki M, Lund JN, Phillips BE: Post-operative electrical muscle stimulation attenuates loss of muscle mass and function following major abdominal surgery in older adults: a split body randomised control trial. Age Ageing 2022, 51(10). 10.1093/ageing/afac234 PMC9621149 36315433

[19] Wollersheim T, Grunow JJ, Carbon NM, Haas K, Malleike J, Ramme SF, Schneider J, Spies CD, Mardian S, Mai K (2019). Muscle wasting and function after muscle activation and early protocol-based physiotherapy: an explorative trial. J Cachexia Sarcopenia Muscle.

[20] Dirks ML, Hansen D, Van Assche A, Dendale P, Van Loon LJ (2015). Neuromuscular electrical stimulation prevents muscle wasting in critically ill comatose patients. Clin Sci (Lond).

[21] Gruther W, Kainberger F, Fialka-Moser V, Paternostro-Sluga T, Quittan M, Spiss C, Crevenna R (2010). Effects of neuromuscular electrical stimulation on muscle layer thickness of knee extensor muscles in intensive care unit patients: a pilot study. J Rehabil Med.

[22] Fischer A, Spiegl M, Altmann K, Winkler A, Salamon A, Themessl-Huber M, Mouhieddine M, Strasser EM, Schiferer A, Paternostro-Sluga T (2016). Muscle mass, strength and functional outcomes in critically ill patients after cardiothoracic surgery: does neuromuscular electrical stimulation help? The Catastim 2 randomized controlled trial. Crit Care.

[23] Kho ME, Truong AD, Zanni JM, Ciesla ND, Brower RG, Palmer JB, Needham DM (2015). Neuromuscular electrical stimulation in mechanically ventilated patients: a randomized, sham-controlled pilot trial with blinded outcome assessment. J Crit Care.

[24] Gutierrez-Arias RE, Zapata-Quiroz CC, Prenafeta-Pedemonte BO, Nasar-Lillo NA, Gallardo-Zamorano DI (2021). Effect of neuromuscular electrical stimulation on the duration of mechanical ventilation. Respir Care.

[25] Campos DR, Bueno TBC, Anjos J, Zoppi D, Dantas BG, Gosselink R, Guirro RRJ, Borges MC (2022). Early neuromuscular electrical stimulation in addition to early mobilization improves functional status and decreases hospitalization days of critically ill patients. Crit Care Med.

[26] Banks PA, Bollen TL, Dervenis C, Gooszen HG, Johnson CD, Sarr MG, Tsiotos GG, Vege SS (2013). Acute Pancreatitis Classification Working G: Classification of acute pancreatitis–2012: revision of the Atlanta classification and definitions by international consensus. Gut.

[27] Force ADT, Ranieri VM, Rubenfeld GD, Thompson BT, Ferguson ND, Caldwell E, Fan E, Camporota L, Slutsky AS (2012). Acute respiratory distress syndrome: the Berlin Definition. JAMA.

[28] Dos Santos FV, Cipriano G, Vieira L, Guntzel Chiappa AM, Cipriano GBF, Vieira P, Zago JG, Castilhos M, da Silva ML, Chiappa GR (2020). Neuromuscular electrical stimulation combined with exercise decreases duration of mechanical ventilation in ICU patients: a randomized controlled trial. Physiother Theory Pract.

[29] Sommers J, Engelbert RH, Dettling-Ihnenfeldt D, Gosselink R, Spronk PE, Nollet F, van der Schaaf M (2015). Physiotherapy in the intensive care unit: an evidence-based, expert driven, practical statement and rehabilitation recommendations. Clin Rehabil.

[30] Fontela PC, Glaeser SS, Martins LF, Condessa RL, Prediger DT, Forgiarini SG, Forgiarini LA, Lisboa TC, Friedman G (2021). Medical research council scale predicts spontaneous breathing trial failure and difficult or prolonged weaning of critically ill individuals. Respir Care.

[31] Wieske L, Witteveen E, Verhamme C, Dettling-Ihnenfeldt DS, van der Schaaf M, Schultz MJ, van Schaik IN, Horn J (2014). Early prediction of intensive care unit-acquired weakness using easily available parameters: a prospective observational study. PLoS ONE.

[32] Kiper P, Rimini D, Falla D, Baba A, Rutkowski S, Maistrello L, Turolla A: Does the score on the mrc strength scale reflect instrumented measures of maximal torque and muscle activity in post-stroke survivors? Sensors (Basel) 2021, 21(24). 10.3390/s21248175 PMC8708806 34960269

[33] Pratt J, De Vito G, Narici M, Segurado R, Dolan J, Conroy J, Boreham C (2021). Grip strength performance from 9431 participants of the GenoFit study: normative data and associated factors. Geroscience.

[34] Kawakami R, Murakami H, Sanada K, Tanaka N, Sawada SS, Tabata I, Higuchi M, Miyachi M (2015). Calf circumference as a surrogate marker of muscle mass for diagnosing sarcopenia in Japanese men and women. Geriatr Gerontol Int.

[35] Kawamoto R, Kikuchi A, Akase T, Ninomiya D, Kumagi T (2021). Thigh circumference and handgrip strength are significantly associated with all-cause mortality: findings from a study on Japanese community-dwelling persons. Eur Geriatr Med.

[36] Fei Y, Gao K, Li WQ (2018). Artificial neural network algorithm model as powerful tool to predict acute lung injury following to severe acute pancreatitis. Pancreatology.

[37] Hochman D, Louie B, Bailey R (2006). Determination of patient quality of life following severe acute pancreatitis. Can J Surg.

[38] Sternby H, Mahle M, Linder N, Erichson-Kirst L, Verdonk RC, Dimova A, Ignatavicius P, Ilzarbe L, Koiva P, Penttila A (2019). Mean muscle attenuation correlates with severe acute pancreatitis unlike visceral adipose tissue and subcutaneous adipose tissue. United European Gastroenterol J.

[39] Zhou Y, Hao N, Duan Z, Kong M, Xu M, Zhang D, Xu X, Yuan Q, Li C (2021). Assessment of acute pancreatitis severity and prognosis with CT-measured body composition. Int J Gen Med.

[40] Dinglas VD, Aronson Friedman L, Colantuoni E, Mendez-Tellez PA, Shanholtz CB, Ciesla ND, Pronovost PJ, Needham DM (2017). Muscle weakness and 5-year survival in acute respiratory distress syndrome survivors. Crit Care Med.

[41] Herridge MS, Cheung AM, Tansey CM, Matte-Martyn A, Diaz-Granados N, Al-Saidi F, Cooper AB, Guest CB, Mazer CD, Mehta S (2003). One-year outcomes in survivors of the acute respiratory distress syndrome. N Engl J Med.

[42] Norman K, Stobaus N, Gonzalez MC, Schulzke JD, Pirlich M (2011). Hand grip strength: outcome predictor and marker of nutritional status. Clin Nutr.

[43] Celis-Morales CA, Welsh P, Lyall DM, Steell L, Petermann F, Anderson J, Iliodromiti S, Sillars A, Graham N, Mackay DF (2018). Associations of grip strength with cardiovascular, respiratory, and cancer outcomes and all cause mortality: prospective cohort study of half a million UK Biobank participants. BMJ.

[44] Chen YH, Hsiao HF, Li LF, Chen NH, Huang CC (2019). Effects of electrical muscle stimulation in subjects undergoing prolonged mechanical ventilation. Respir Care.

